# Effects of dietary inclusion of *Moringa oleifera* leaf meal on growth performance of Muscovy ducklings (*Cairina moschata*)

**DOI:** 10.5713/ab.23.0208

**Published:** 2023-11-01

**Authors:** Assem M. Safwat, Luis Sarmiento-Franco, Enass Abd El-khalek, Bahaa M. Abou-Shehema, Osama A. Hassan, Asmaa Sh. Elnaggar

**Affiliations:** 1 Poultry Production Department, Faculty of Agriculture (El-Shatby), Alexandria University, Alexandria 21545, Egypt; 2 Department of Animal Nutrition, Faculty of Veterinary Medicine and Animal Science, University of Yucatan (UADY), Mérida, Yucatán 97100, Mexico; 3 Department of Poultry Nutrition, Animal Production Research Institute, Agriculture Research Center, 21918, Egypt; 4 Department of Animal Production, Faculty of Agriculture, Damanhour University, Damanhour 22512, Egypt

**Keywords:** Antioxidant Status, Digestibility, Ducklings, Immune Response, *Moringa oleifera* Leaf Meal, Productive Performance

## Abstract

**Objective:**

The current experiment was performed to investigate the influence of different dietary levels of *Moringa oleifera* leaf meal (MOLM) on productive performance, nutrient digestibility, blood parameters, immune response, caecal microbiota, and carcass characteristics of Muscovy ducks (*Cairina moschata*) during 7 to 63 d of age.

**Methods:**

A total of 240 unsexed 7-d-old ducklings were distributed into five (treatment) groups; each one contained six replicates with eight ducklings each. Birds of the first group were fed basal diet without MOLM and served as control, while the other four groups were fed basal diet with 0.25%, 0.50%, 1.0%, and 2.0% MOLM inclusion level, respectively.

**Results:**

The obtained results revealed that including MOLM in the diets significantly improved body weight, body weight gain, feed conversion ratio and economic efficiency compared with the control group. Among the different MOLM inclusion treatments, increasing MOLM inclusion level decreased (p<0.05) such previous parameters. Decreasing MOLM inclusion levels in duckling diets increased (p>0.05) the digestibility of organic matter, crude protein, ether extract, and nitrogen free extract, however all MOLM treatments were significantly higher than the control group. Results also revealed that feeding ducks lower MOLM inclusion levels (0.25% or 0.50%) improved blood parameters (p<0.05) compared with the higher inclusion levels (1.0% or 2.0% MOLM) and the control group. Ducks fed different MOLM levels had significantly higher phagocyte index and activity, immunoglobulin G (IgG), IgM, total antioxidant capacity, glutathione peroxidase activity, and superoxide dismutase activity compared with control group.

**Conclusion:**

Despite the beneficial effects of all MOLM treatments on growth performance, nutrient digestibility, physiological status, and immune response of duckling, the increasing MOLM inclusion level in the diet had deleterious effects on such studied traits, consequently 0.25% was the best MOLM inclusion level in duckling diets.

## INTRODUCTION

Both scientific and commercial poultry nutrition sectors are investigating a variety of growth promoters as safe antibiotic alternatives for enhancing poultry health and growth [1]. The application of medicinal herbs and various plant extracts has recently gained more attention as replacements for antibiotics as natural growth promoters, especially with increasing the demand for organic poultry products [2]. One of the abundant medicinal plants that could be utilized to improve production performance and enhance the immune system of poultry, without an additional cost for synthetic antibiotics, is *Moringa oleifera* plant [3].

*Moringa oleifera* is a well-known cultivated species which grows in tropical and subtropical regions. It is regarded as a miracle tree due to its abundant supply of various nutrients with high biological value [2]. The leaves of *M. oleifera* are distinguished by their impressive nutritional and medicinal properties, thus *M. oleifera* leaf meal is used as a phytogenic growth promoter and immune enhancer due to its antioxidant, antimicrobial, and hypocholesterolemic effects on different livestock species [4]. It contains high levels of essential amino acids (e.g., methionine, lysine, threonine, tryptophan, and valine), fatty acids (such as, α-linolenic, palmitic, and capric acid), vitamins (including, pro-vitamin A as beta-carotene, vitamin B complex, vitamin C, and vitamin E), as well as various inorganic minerals [5]. Furthermore, Stohs and Hartman [3] reported that *M. oleifera* contains an active component known as niaziridin which can improve the absorption of various nutrients, vitamins, and minerals in the host’s gastrointestinal tract.

Moreover, *M. oleifera* leaves contain a variety of phytonutrient compounds, such as polyphenols, tannins, glycosides, carotenoids, tocopherols, and ascorbic acid that exhibits antioxidant properties by activating antioxidant enzymes and transforming oxygen-free radical to nonradical compounds [6]. In addition, *Moringa* leaf extracts possess antibacterial and antifungal functions due to the presence of different lipophilic metabolites (carboxylic acid, chitinases, isothiocyanates, glycoside cyanides) in the plant cell walls [7]; such phytochemicals in *M. oleifera* leaves might play an important role in enhancing immune response and improving health status of poultry species [8]. In this connection, Castillo et al [9] found that *Moringa oleifera* leaf flour substantially increased the immunity in Japanese quail by inhibiting the growth of both Gram-positive and Gram-negative bacteria.

There is a wide range of *Moringa oleifera* leaf meal (MOLM) inclusion levels in poultry diets. For instance, Dey and De [10] found that 0.25% or 0.40% MOLM in broiler diets represented significant improvement in body weight (BW) compared to the control group. Meanwhile, Olugbemi et al [11] supplemented a maximum level of MOLM (5%) with no adverse effects on growth performance and feed conversion ratio (FCR) of broilers. These findings confirmed the fact that feeding moringa leaves had no deleterious effects on normal physiology and growth in the experimental birds.

Despite the favorable results reported by several studies on the use of *M. oleifera* leaves as a poultry feed ingredient, the optimal inclusion level in ducking rations and their mode of action are still under consideration [2]. Therefore, the current study aimed to investigate the influence of different dietary inclusion levels of MOLM on growth performance, nutrient digestibility, cecal bacterial count, blood constituents, immune response, and carcass characteristics of ducklings during 7 to 63 d of age.

## MATERIALS AND METHODS

### Animal care

The experimental procedures were carried out according to the Institutional Animal Care and Use Committee (IACUC) of Damanhour University, Egypt; with approval code: DUFA-2021-3.

### Study site

This study was conducted at El-Bostan Farm, Faculty of Agriculture, Damanhour University, and the laboratory analyses were carried out at Faculty of Agriculture (El-Shatby), Alexandria University and El-Sabahia Poultry Research Station, Animal Production Research Institute, Agricultural Research Center, Egypt.

### *Moringa* leaf meal preparation

*M. oleifera* leaves were harvested from a private commercial farm in Egypt. The leaves were separated from branches and dried in air oven at 60°C to 70°C for two days. Thereafter, the dried leaves were ground with a hammer mill (3.0 mm sieve) to make the MOLM which was included in the experimental diets. The chemical composition of MOLM used in the current study is presented in Table 1.

### Birds, management, and experimental design

A total number of 240 unsexed 7-d-old Muscovy ducklings (*Cairina moschata*), with an average initial BW of 135±8 g, were used. The ducklings were individually weighed, and randomly distributed into five treatment groups of six replicates with eight ducklings each. The ducklings were reared in 30 floor pens (1.0×1.75×2.0 m each) in an open-sided system house and raised under the same sanitary and management settings. They were allocated to the following dietary treatments: the first group was fed the basal diet without MOLM inclusion (control), while 2nd, 3rd, 4th, and 5th group were fed basal diet included with different MOLM levels by 0.25%, 0.50%, 1.0%, and 2.0%, respectively. The experimental diets were formulated to meet the nutrient requirements of ducklings according to NRC [12]. The ingredients and nutrient composition of diets fed during starting and growing periods are illustrated in Table 2. Feed and water were provided *ad-libitum* throughout the experimental period. Birds were subjected to a light regimen with 23 hours of light during the first week and 20 hours of light from the second week until the end of the fattening period. All ducklings were brooded at 33°C, upon arrival, temperature then declined gradually to 30-27 and 24°C during the second week and from 3 to 9 weeks of age, respectively.

### Productive performance

Performance parameters including ducklings live BW and body weight gain (BWG) were individually determined, while feed intake (FI) and FCR were recorded weekly for each replicate within treatment. The European production efficiency index (EPEI) was calculated according to Marcu et al [13]. The price of ducks and experimental diets were calculated according to the cost of the native market at the timing of the experiment. The economic efficiency was represented as (net revenue/total cost) ×100 while, relative economic efficiency (REE) was calculated as (economic efficiency of a treatment / economic efficiency of the control)×100.

### Digestibility trial

At the end of the experimental period (at 63 d of age), a digestibility trial was conducted using 30 drakes around the treatment average (six from each treatment) to determine the apparent nutrients digestibility for the experimental diets. Birds were housed in metabolic cages (42×50×50 cm/cage) for 11-d trial (7 d of adaptation period + 4 d of collection period). Feed consumption and total excreta output from each bird were accurately determined during the collection period. The crude protein (CP), crude fiber (CF), ether extract (EE), and ash content of dried excreta as well as those of experimental diets were determined following the AOAC [14] procedures. The apparent CP digestibility was calculated after correcting for uric acid nitrogen in the excreta. The amount of nitrogen free extract (NFE) on a dry matter (DM) basis was calculated by subtracting the sum of CP, CF, EE, and ash percentages from hundred.

### Intestinal bacterial count

During slaughter, six samples of intestinal content (from ileum) were collected from each treatment for enumeration of total bacterial count (TBC), *Escherichia coli*, *Proteus* spp., and *Lactobacillus* spp. as a colony forming unit (CFU) using modified methods described by Baurhoo et al [15] that differed only in the agars used.

### Blood collection and hematobiochemical analyses

At the end of the experimental period, six blood samples (about 3 mL) from each treatment (a sample per replicate) were collected from the brachial vein. Each sample was divided into two parts, the first part was retained without heparin to obtain serum and the second part was kept in heparinized tube to estimate the complete blood count test. Serum was separated by centrifuging the blood samples at 4,000 rpm for 15 min, then stored at −20°C until the biochemical analysis.

Red blood cells (RBCs 10^6^/mm^3^), white blood cells (WBCs 10^3^/mm^3^), the differential count, hemoglobin concentration (Hb) and packed cell volume percentage (PCV %) were determined. Glucose, total protein, albumin, total lipids, triglyceride (TG), cholesterol, high density lipoprotein (HDL), low density lipoprotein (LDL), aspartate aminotransferase (AST), alanine aminotransferase (ALT), uric acid, creatinine, total antioxidant capacity (TAC), glutathione peroxidase (GSH-PX), superoxide dismutase (SOD), malondialdehyde (MDA), tri-iodothyronine (T_3_), and thyroxin (T_4_) were determined using specific kits obtained from sentinel CH Milano, Italy; CAL-TECH Diagnostics, Inc., Chino, CA, USA; by means of a spectrophotometer (Beckman DU-530, Hanau, Germany), Diagnostic Products Corporation, Los Angeles, USA; or Reactivos GPL, Barcelona, Spain; according to kits manufacturers recommendations. The enzyme-linked immune-sorbent assay (ELISA) technique was performed to assess immunoglobulin fractions concentrations (IgG and IgM) using commercial kits (Bethyl Laboratories, Montgomery, TX, USA; Catalog No. E33-104-200218 and E33-102-180410, respectively). The method of Leijh et al [16] was used to determine the phagocyte index and activity (PI and PA). Serum bactericidal activity (BA) to *Aeromonas hydrophilia* strain was determined according to Rainger and Rowley [17]. The lymphocyte transformation test (LTT) was carried out following the method described by Balhaa et al [18].

### Carcass characteristics

At the end of the experiment (63 d of age), six ducks from each treatment were selected, overnight fasted, individually weighed as pre-slaughter weight, and slaughtered. After complete bleeding, feather picking, and evisceration; the carcass, internal organs (liver, gizzard, pancreas, spleen, and thymus gland), as well as abdominal fat were separately weighed. The percentages of empty carcasses and organs were calculated based on the pre-slaughter weight.

### Statistical analysis

Data were analysed in a completely randomized design using the general linear model procedure of SAS program 9.4 (SAS Inst. Inc. Cary, NC, USA). The percentage data of the studied traits were transformed to square root or arc sine, while bacterial counts were converted using Log transformation before statistical analysis. The experimental unit for each studied parameter was the replicate. The statistical model used was:


$${\text{Y}}_{\text{ij}}=\mu +{\text{T}}_{\text{i}}+{\text{e}}_{\text{ij}}$$

Where; Y_ij_ = an observation, μ = overall mean, T_i_ = effect of treatment (i = 1,2, ….5) and e_ij_ = experimental random error. The difference among means was determined using Duncan’s new multiple range test [19], and the significance was considered at p≤0.05.

## RESULTS

### Productive performance traits

The productive performance, production index, and economic efficiency of ducks fed basal diet included with graded levels of MOLM during 7 to 63 d of age are shown in Table 3. It can be observed that initial body weight at 7th day of age did not represent any significant difference among the experimental groups. Results indicated that ducks fed basal diet with MOLM at different inclusion levels improved (p<0.05) BW, BWG, FCR, economic efficiency, REE, and EPEI compared with the control group. Although among MOLM treatments, it could be noticed that all performance traits ware improved by decreasing MOLM inclusion level, with the best values (p<0.05) achieved by the lower MOLM inclusion treatments (0.25% and 0.50%) compared with the higher inclusion groups (1.0% and 2.0%). Additionally, the amount of FI was increased with increasing MOLM inclusion level in duckling diets, while the control group significantly consumed the highest amount of feed. The technical evaluation expressed as EPEI in the present study (Table 3) clearly indicated that including different levels of MOLM in duckling diets significantly produced better EPEI values compared with the control group.

### Apparent nutrients digestibility and intestinal bacterial count

The digestibility coefficient values of organic matter, CP, EE, and NFE for all MOLM treatments were significantly higher than the control group (Table 4). Also, it could be observed that increasing MOLM inclusion level in the diet significantly decreased CP digestibility coefficient, with the highest values (p<0.05) associated with the groups fed diets included with 0.25% and 0.50% MOLM compared with the other experimental groups. However, digestibility coefficient values of CF did not represent any statistical difference among the experimental groups. Also, it was showed in Table 4 that feeding different levels of MOLM significantly reduced the TBC, *E. coli*, and *Proteus* count compared with the control group. The opposite trend was observed for *lactobacillus* sp. count which increased with MOLM treatments (p<0.05). Among MOLM treatments, there was an insignificant increasing trend of TBC when MOLM was increased in the diet.

### Hematological parameters and immunological indices

Data of Table 5 illustrated that RBCs, Hb, and PCV for ducks fed diets included with 0.25% and 0.50% MOLM were increased (p<0.05) compared with the other experimental treatments. In the same line, ducks fed basal diet included with 0.25%, 0.50%, and 1.0% MOLM had significantly higher values of WBCs and lymphocytes compared with birds fed basal diet with 2.0% of MOLM and control groups. However, the heterophil percent and heterophil/lymphocyte ratio for all MOLM treated groups did not represent any statistical difference among the experimental groups.

Also, results of Table 5 revealed that feeding ducks on different levels of MOLM improved immunological indices (p<0.05). Since PA, PI, BA, LTT, and immunoglobulins (IgG and IgM) for birds of all experimental MOLM groups were significantly higher than ducks fed the control diet, without any statistical difference between MOLM treated groups concerning these previous indices.

### Biochemical constituents of blood

Data of Table 6 indicated that values of total protein, globulin, and TGs for birds fed different levels of MOLM were higher (p<0.05) than the control group. On the other hand, glucose, total lipid, cholesterol, and LDL were significantly decreased for groups fed MOLM levels compared to the control group. Concerning moringa treatments, the groups fed 0.25%, 0.50%, and 1.0% MOLM had significantly better measurements than the group of 2.0% MOLM regarding total protein, globulin, total lipid, cholesterol, and LDL.

Additionally, feeding ducks on different levels of MOLM significantly decreased uric acid, creatinine, AST, ALT, and MDA compared with the control group. The opposite trend was observed for TAC, GSH-PX, SOD activity, and T_3_ which increased (p<0.05) with MOLM treatments. While there was a significant deterioration trend among MOLM treatments regarding creatinine, TAC, MDA, and T_3_ concentrations when MOLM inclusion level increased in duckling diets. However, T_4_/T_3_ ratio did not show any significant difference among all experimental groups with an increasing trend when MOLM inclusion level increased in the diet.

### Carcass characteristics

The results in Table 7 reveal that the percentages of dressing and total edible parts in ducks fed different inclusion levels of MOLM were significantly higher than in the control group. However, the opposite trend was observed for abdominal fat percentage. Furthermore, there was a significant improvement concerning these mentioned traits as MOLM inclusion level decreased in the diet, with the best values (p<0.05) recorded for the groups fed 0.25% and 0.50% MOLM compared with the higher inclusion groups. Moreover, liver, gizzard, pancreas, spleen, and thymus percentages did not differ statistically among the experimental groups.

## DISCUSSION

The substantial increase in BW and BWG for ducklings fed basal diet supplemented with different MOLM levels may be primarily attributable to the considerable amount of essential nutrients, vitamins, and minerals found in *Moringa* leaves [20]. The observed beneficial effect with decreasing MOLM inclusion level on BW and BWG may be attributed to the optimum levels of different bioactive compounds presented in the low MOLM levels (0.25% and 0.50%) that may play a role in improving nutrient utilization efficiency of those groups [21]. In this regard, Gheisar et al [22] concluded that addition of blend phytogenic to ducks’ diet improved BWG. Consistent with the obtained results, Dey and De [10] found that 0.25% or 0.40% MOLM in broiler diets represented significant improvement in BW compared to the control group.

The decrease in FI for ducklings fed different levels of MOLM could be attributed to the lower palatability of these diets, particularly as MOLM inclusion levels increased in the diet (1.0% and 2.0%). Consequently, the unpalatability nature of a feedstuff prevents the chick from consuming an adequate quantity of the feed as reported by Attia [23]. Furthermore, it was believed that moringa contains different anti-nutritional factors, such as tannins and phytates (by 1.9% and 2.54% of DM, respectively), which many negatively affect (at high concentrations) feed utilization [2]. The current results of FCR improvements for ducklings fed different levels of MOLM coincided with the previous results of Dey and De [10] who explained that as it was a result of decreasing FI and increasing BWG. Although Makanjuola et al [24] reported that using 0.20%, 0.40%, and 0.60% of MOLM in broiler diets did not significantly influence the FI or FCR, that could be attributed to the lower MOLM inclusion levels used in that study. The resulting improvement in EPEI values for ducklings fed diets included with different levels of MOLM could be explained on the light of FCR and BWG improvements.

The improvement in digestion coefficient of nutrients may be since MOLM stimulates the secretion of some digestive enzymes (i.e., pepsin, protease, lipase, and amylase) that improve nutrients digestion and consequently BWG and FCR [23].

The significant decrease of cecal TBC, *E. coli*, and *Proteus* counts as well as the increase of *Lactobacillus* sp. with including MOLM in the duckling diets are keeping with previous results of Yang et al [25] who stated that *M. oleifera* significantly improved intestinal status by increasing Lactobacillus counts in the ileum and decreasing *E. coli*, thereby enhancing broiler immunity. According to Sanchez et al [26], intestinal microbiota of animals has positive effects on maintaining the intestinal epithelial integrity and barrier, immunomodulation, production of neurotransmitters which can support the nutrition and the health of the host animals. The extracts derived from *M. oleifera* leaves possess antibacterial and antifungal properties against a variety of bacterial and fungal species [27]. In this regard, Pandey et al [28] highlighted that *M. oleifera* contains benzyl carbamate, benzyl isothiocyanates and benzyl thiocarboxamide which act as antimicrobial agents. Additionally, Jabeen et al [7] mentioned that the antimicrobial properties of *Moringa oleifera* may be due to the presence of lipophilic compounds and various metabolites (e.g., carboxylic acid, 2,4-diacetyl phloroglucinol, enzymes, and chitinases) in plant cell walls. Bhumika and Bijal [29] showed that solvent extracts of *M. oleifera* components (leaves, flower, pulp, and seed) have bactericidal effect against *E. coli* and *S. aureus*.

The significant improvements of RBCs, Hb, PCV, WBCs, and lymphocytes as affected by dietary MOLM inclusion levels are consistent with earlier findings [30]. Red blood cells are responsible for transporting oxygen and carbon dioxide in the blood as well as producing hemoglobin, hence higher RBCs values indicate a greater potential for such functions and a better health status. Higher hematological parameter values may be attributed to MOLM protein content, as MOLM has a blood-boosting effect.

The observed improvement in immunological indices as a result of including different levels of MOLM in ducking diets could be due to the presence of biochemical agents in *M. oleifera* plant which have immune-stimulant activities. According to Khan et al [31], the bioactive components in *M. oleifera* may boost the number of B lymphocytes, which are responsible for antibody production, leading to a rise in antibody titer. MOLM also contains a number of biochemical components, including quercetin, isothiocyanate, and kaempferol glucosides which have anti-inflammatory activities [3]. Moreover, the presence of various proteins and peptides, such as isothiocyanates and glycoside cyanides, in *M. oleifera* leaf extracts were able to positively alter the immunological response and activate macrophage-induced phagocytosis [32]. Furthermore, previous studies have also established the immune functions of *M. oleifera* [27].

The favorable effect of dietary *M. oleifera* inclusion on ducking lipid profile could be related to the antioxidant compounds presented in *Moringa* including the flavonoid mevalonic acid that produced from acetyl-CoA, such compounds reduce cholesterol formation through inhibiting the activity of the 3-methyl, 3-hydroxy-glutaryl-CoA reductase enzyme, which converts acetyl-CoA to mevalonic acid [33]. Additionally, *M. oleifera* leaf extracts are reported to possess a hypocholesterolemic function as *M. oleifera* leaves contain moringine and moringinine components that were identified as anti-hypoglycemic agents [34]. The significant increase in blood protein concentrations may be attributed to the antioxidant compounds presented in *M. oleifera* leaf, which inhibit corticosterone release and reduce protein catabolism, resulting in higher plasma protein concentrations. In line with the current result, a linear increase of plasma protein with increasing MOLM inclusion level has been previously documented by Hassan et al [35]. Concerning the effect of MOLM on decreasing plasma glucose level, Jaiswal et al [36] suggested that *Moringa oleifera* stimulates the action of insulin-like growth factor I (IGF-I) which in turn decreases gluconeogenesis and increases glucose uptake by cells.

The significant decrement of ALT and AST levels as well as creatinine and uric acid concentrations, at the normal range, for the ducks fed diets containing MOLM may reflect an improvement in liver and kidney functions. The role of MOLM supplementation in AST and ALT improvement could be due to the presence of polyphenols or their metabolites in MOLM, which have high tissue bio-efficacy. The improvement of TAC, GSH-PX, SOD activity in ducks fed diets containing MOLM was attributed to the higher concentration of various phytochemicals that act as antioxidant compounds such as carotenoids, polyphenols, ascorbic acid, and tocopherol [4,8], meanwhile the flavonoids in MOLM may act as radical scavengers that decrease MDA concentration [8]. Such biochemical active metabolites found in MOLM are responsible for the improvement of health status and lead to better productive performance in the experimental ducklings fed MOLM.

The obvious increases in the percentages of dressing and total edible parts for experimental ducklings fed diets included with MOLM may be attributed to the high nutrients’ digestibility and bioavailability of diets containing MOLM, which resulted in better growth and development of body organs. In this context, Olugbemi et al [11] reported that *Moringa oleifera* is a rich source of amino acids, vitamins, and micronutrients. Additionally, *M. oleifera* leaves have a high percentage of available protein (e.g., pepsin soluble nitrogen) and a low level of acid detergent insoluble protein [37]. On the other hand, the observed reduction in carcass abdominal fat could be due to the hypolipidemic effect of *M. Oleifera* leaf meal as well as the effect of the antioxidant compounds such as ascorbic acid and tocopherol. The favorable effects of MOLM inclusion on carcass characteristics were reported by Mousa et al [38] who included *M. oleifera* by different levels and found that a 5% inclusion level in broiler diets increased the percentage of carcass yield.

## CONCLUSION

It can be concluded that increasing MOLM inclusion level (within the studied range of 0.25% to 2.0%) had no significant unfavorable effects on duckling growth performance, nutrient digestibility, intestinal bacterial count, physiological status, immune response, and economic efficiency, however the best results were with the 0.25% MOLM and therefore it is the recommended inclusion level in duckling diets. Although, all studied MOLM inclusion levels (up to 2.0%) were better than the control group regarding such studied traits.

## Figures and Tables

**Table 1:** Chemical analysis of the experimental *Moringa oleifera* leaf meal

Component	%
Dry matter	95.20
Crude protein	21.90
Crude fiber	12.94
Ether extract	3.16
Nitrogen free extract	50.38
Ash	6.82
Total polyphenols	3.91
Tannins	1.90
Phytate	2.54

**Table 2:** Composition and chemical analysis of the experimental basal diets containing different levels of *Moringa oleifera* leaf meal for growing ducks during starter and grower period

Items	Starter (7 to 35 d)					Grower (36 to 63 d)				
	
	Control	MOLM levels (%)				Control	MOLM levels (%)			
	
		0.25	0.50	1.0	2.0		0.25	0.50	1.0	2.0
Ingredient (%)										
MOLM	-	0.25	0.50	1.00	2.00	-	0.25	0.50	1.00	2.00
Yellow corn	56.45	56.20	56.25	55.50	54.65	68.00	67.8	67.45	67.00	66.35
Soybean meal (44%)	38.30	38.20	38	37.90	37.5	26.70	26.6	26.55	26.35	25.75
Sunflower oil	1.45	1.55	1.45	1.80	2.05	1.50	1.55	1.7	1.85	2.10
Limestone	1.00	1.00	1.00	0.98	0.97	1.00	1.00	1.00	0.98	0.97
Dicalcium phosphate	2.00	2.00	2.00	2.00	2.00	2.00	2.00	2.00	2.00	2.00
Salt (NaCl)	0.30	0.30	0.30	0.30	0.30	0.30	0.30	0.30	0.30	0.30
Vit+Min premix^1)^	0.30	0.30	0.30	0.30	0.30	0.30	0.30	0.30	0.30	0.30
DL-Methionine	0.10	0.10	0.10	0.12	0.13	0.10	0.10	0.10	0.12	0.13
Anti-coccidia	0.10	0.10	0.10	0.10	0.10	0.10	0.10	0.10	0.10	0.10
Total	100	100	100	100	100	100	100	100	100	100
Chemical composition (% on DM basis)										
ME (kcal/kg)^2)^	2,893	2,890	2,887	2,885	2,880	3,024	3,020	3,021	3,018	3,012
Crude protein^3)^	22.18	22.17	22.15	22.14	22.10	18.09	17.97	18.02	18.07	17.95
Crude fiber^3)^	4.25	4.27	4.29	4.33	4.39	3.56	3.58	3.60	3.63	3.68
Ether extract^3)^	4.23	4.32	4.27	4.56	4.76	4.55	4.60	4.68	4.82	4.94
Lysine^2)^	1.18	1.17	1.19	1.18	1.17	0.89	0.89	0.90	0.87	0.88
Methionine^2)^	0.45	0.44	0.43	0.46	0.47	0.39	0.38	0.38	0.40	0.41
Methionine+cystine^2)^	0.79	0.80	0.78	0.79	0.80	0.69	0.68	0.69	0.70	0.71
Threonine^2)^	0.89	0.89	0.88	0.87	0.87	0.67	0.67	0.66	0.66	0.65
Tryptophan^2)^	0.37	0.36	0.36	0.35	0.34	0.27	0.26	0.26	0.25	0.25
Calcium^2)^	0.93	0.92	0.93	0.94	0.96	0.94	0.92	0.95	0.96	0.98
Available phosphorus^2)^	0.45	0.44	0.46	0.44	0.45	0.39	0.38	0.38	0.37	0.38

MOLM, *Moringa oleifera* leaf meal; DM, dry matter; ME, metabolizable energy.

1) Vit+Min premix. Provided per kilogram of the diet: vit. A, 6,000 IU; vit. E (dl-α-tocopherol acetate, 10 IU; menadione, 2.5 mg; vit. D_3_, 2,000 ICU; riboflavin, 2.5 mg; calcium pantothenate, 10 mg; nicotinic acid, 12 mg; Choline chloride, 300 mg; vit. B_12_, 4 μg; vit. B_6_, 5 mg; thiamine, 3 mg; folic acid, 0.50 mg; and biotin, 0.02 mg; Mn, 80 mg; Zn, 60 mg; Fe, 35 mg; Cu, 8 mg; Se, 0.1 mg.

2) Calculated.

3) Analyzed.

**Table 3:** Influence of different dietary inclusion levels of *Moringa oleifera* leaf meal on growth performance and economic parameters of ducklings during 7–63 d of age

Items	Control	MOLM levels (%)				SEM	p-value

		0.25	0.50	1.0	2.0		
Initial body weight (g)	134.0	136.0	134.0	136.0	135.0	1.141	0.9000
Final body weight (g)	3,000^c^	3,765^a^	3,596^a^	3,293^b^	3,200^b^	28.98	0.0003
Body weight gain (g)	2,866^c^	3,630^a^	3,462^a^	3,157^b^	3,065^b^	10.90	0.0004
Feed consumption (g)	9,100^a^	8,000^c^	8,400^b^	8,500^b^	8,600^b^	31.33	0.0004
Feed conversion ratio (g feed/g gain)	3.18^a^	2.20^c^	2.43^c^	2.69^b^	2.88^b^	0.050	0.0001
EE (%)	61^c^	91^a^	90^a^	81^b^	78^b^	3.022	0.0001
REE (%)	100^c^	148^a^	146^a^	131^b^	126^b^	9.104	0.0001
EPEI (%)	76.8^c^	115.2^a^	118.9^a^	103.0^b^	99.5^b^	3.502	0.0002

MOLM, *Moringa oleifera* leaf meal; SEM, standard error of mean; EE, economic efficiency; REE, relative economic efficiency; EPEI, European production efficiency index.

a–c Means in the same row followed by different letters are significantly different at (p<0.05).

**Table 4:** Influence of different dietary inclusion levels of *Moringa oleifera* leaf meal on apparent nutrients digestibility and intestinal bacterial count of ducklings during 7 to 63 d of age

Items	Control	MOLM levels (%)				SEM	p-value

		0.25	0.50	1.0	2.0		
Apparent nutrients digestibility (%)							
Organic matter	69.9^b^	73.67^a^	70.90^a^	72.90^a^	71.2^a^	3.092	0.0001
Crud protein	65.53^c^	73.14^a^	72.66^a^	70.04^ab^	68.39^b^	2.963	0.0001
Ether extract	64.52^b^	74.37^a^	71.09^a^	69.93^a^	70.29^a^	6.321	0.0002
Crud fiber	35.14	39.62	40.26	42.88	38.79	1.172	0.0870
Nitrogen free extract	66.34^b^	74.42^a^	75.81^a^	75.14^a^	73.03^a^	2.849	0.0001
Intestinal bacterial count							
TBC (CFU ×10^6^)	3.02^a^	2.12^b^	2.27^b^	2.32^b^	2.47^b^	0.0753	0.0005
*E. Coli* (CFU ×10^3^)	1.35^a^	0.998^b^	0.851^b^	0.752^b^	0.751^b^	0.0452	0.0003
*Proteus* (CFU ×10^3^)	0.998^a^	0.452^b^	0.566^b^	0.423^b^	0.451^b^	0.0245	0.0002
*Lactobacillus* spp. (CFU ×10^3^)	1.52^b^	2.62^a^	2.57^a^	2.62^a^	2.87^a^	0.1481	0.0003

MOLM, *Moringa oleifera* leaf meal; SEM, standard error of mean; TBC, total bacterial count; CFU, colony forming unit; *E. Coli*, *Escherichia coli*.

a–c Means in the same row followed by different letters are significantly different at (p<0.05).

**Table 5:** Influence of different dietary inclusion levels of *Moringa oleifera* leaf meal on hematological parameters and some immunological indices of ducklings during 7 to 63 d of age

Items	Control	MOLM levels (%)				SEM	p-value

		0.25	0.50	1.0	2.0		
RBCs (10^6^/mm^3^)	2.37^b^	2.65^a^	2.67^a^	2.45^b^	2.45^b^	0.0203	0.0001
Hb (g/100 mL)	10.51^c^	13.71^a^	14.50^a^	11.41^b^	11.22^b^	0.1611	0.0006
PCV (%)	30.50^c^	37.00^a^	36.51^a^	33.30^b^	32.50^b^	0.6180	0.0002
WBCs (10^3^/mm^3^)	22.90^b^	28.75^a^	27.10^a^	28.45^a^	21.50^b^	0.1732	0.0001
Lymphocytes (%)	42.83^b^	47.22^a^	48.72^a^	49.77^a^	43.70^b^	0.1953	0.0002
Heterophils (%)	24.14	22.20	21.41	20.00	26.72	0.1360	0.0952
H/L ratio	0.596	0.536	0.513	0.564	0.541	0.0051	0.0934
PA (%)	18.52^b^	25.90^a^	22.21^a^	23.80^a^	24.50^a^	0.2242	0.0001
PI (%)	17.90^b^	26.52^a^	25.91^a^	24.50^a^	24.99^a^	0.1263	0.0004
BA	30.52^c^	40.00^a^	42.52^a^	41.30^a^	35.55^b^	0.3074	0.0004
LTT	19.54^b^	24.20^a^	23.45^a^	26.55^a^	26.00^a^	0.1953	0.0005

MOLM, *Moringa oleifera* leaf meal; SEM, standard error of mean; RBCs, red blood cells count; Hb, hemoglobin; PCV, packed cell volume; WBCs, White blood cell count; PA, phagocytic activity; PI, phagocytic index; BA, bactericidal activity; LTT, lymphocyte transformation test.

a–c Means in the same row followed by different letters are significantly different at (p<0.05).

**Table 6:** Influence of different dietary inclusion levels of *Moringa oleifera l*eaf meal on biochemical blood constituents of ducklings during 7 to 63 d of age

Items	Control	MOLM levels (%)				SEM	p-value

		0.25	0.50	1.0	2.0		
Glucose (mg/L)	2,200^a^	1,900^b^	1,810^b^	1,840^b^	1,600^c^	15.74	0.0002
Total protein (g/L)	59.00^c^	67.80^a^	67.00^a^	65.50^a^	59.80^b^	0.460	0.0002
Albumin (g/L)	33.90	33.30	34.50	33.40	30.30	0.681	0.0801
Globulin (g/L)	25.10^c^	34.50^a^	32.50^a^	32.10^a^	29.60^b^	0.793	0.0001
Total lipid (mmol/L)	8.71	7.70^c^	7.61^c^	7.66^c^	8.07^b^	0.243	0.0002
TG (mmol/L)	0.87^b^	1.02^a^	1.04^a^	1.04^a^	1.06^a^	0.094	0.0003
Chol. (mmol/L)	3.59^a^	2.77^c^	2.66^c^	2.69^c^	8.08^b^	0.361	0.0064
HDL (mmol/L)	0.94	1.20	1.23	1.17	1.21	0.057	0.0754
LDL (mmol/L)	2.16^a^	1.60^c^	1.53^c^	1.57^c^	1.73^b^	0.132	0.0003
AST (U/L)	65.53^a^	51.70^b^	50.10^b^	52.55^b^	53.22^b^	0.6691	0.0001
ALT (U/L)	30.67^a^	23.13^b^	27.87^b^	22.60^b^	23.71^b^	0.3163	0.0001
Uric acid (μmol/L)	184.39^a^	149.89^b^	173.09^b^	136.80^b^	143.94^b^	14.361	0.0007
Creatinine (μmol/L)	163.58^a^	88.15^c^	96.38^b^	96.38^b^	103.45^b^	8.690	0.0001
TAC (mmol/L)	1.35^b^	1.99^a^	2.01^a^	2.06^a^	2.13^a^	0.0580	0.0004
GSH-PX (U/L)	3.35^b^	4.99^a^	4.74^a^	4.85^a^	4.55^a^	0.1953	0.0003
SOD (U/ml)	2.41^b^	2.49^a^	2.51^a^	2.51^a^	2.50^a^	0.2721	0.0006
MDA (μmol/L)	77.00^a^	50.00^b^	55.00^b^	60.00^b^	68.00^b^	0.5696	0.0001
T_3_ (mg/L)	20.80^c^	32.20^a^	31.00^a^	29.90^b^	29.50^b^	0.0662	0.0002
T_4_ (mg/L)	40.53^b^	60.54^a^	60.99^a^	60.89^a^	60.82^a^	0.0603	0.0001
T_4_/T_3_ ratio	1.95	1.88	1.97	2.04	2.06	0.067	0.066
IgG (mg/L)	440.5^b^	485.5^a^	488.0^a^	486.5^a^	487.0^a^	11. 931	0.0001
IgM (mg/L)	223.0^b^	240.0^a^	236.0^a^	239.0^a^	240.0^a^	9.285	0.0007

MOLM, *Moringa oleifera* leaf meal; SEM, standard error of mean; TG, triglycerides; Chol., total cholesterol; HDL, high density lipoprotein; LDL, low density lipoprotein; AST, aspartate amino transferase; ALT, alanine amino transferase; TAC, total antioxidant capacity; GSH-PX, glutathione peroxidase; SOD, superoxide dismutase; MDA, malondialdehyde; T_3_, triiodothyronine; IgG, immunoglobulin G; IgM, immunoglobulin M.

a–c Means in the same row followed by different letters are significantly different at (p<0.05).

**Table 7:** Influence of different dietary inclusion levels of *Moringa oleifera* leaf meal on carcass characteristics, relative abdominal fat, and organ weight of ducklings during 7 to 63 d of age

Traits (%)	Control	MOLM levels (%)				SEM	p-value

		0.25	0.50	1.0	2.0		
Dressing	61.75^c^	71.45^a^	70.09^a^	67.39^b^	66.87^b^	1.721	0.0003
Total edible parts	68.84^c^	76.90^a^	75.70^a^	71.00^b^	70.92^b^	1.760	0.0001
Liver	1.98	2.09	1.93	2.24	2.23	0.0612	0.0947
Gizzard	2.58	3.02	3.17	3.54	3.08	0.0913	0.0992
Pancreas	0.316	0.355	0.334	0.353	0.359	0.0151	0.2921
Abdominal fat	0.964^a^	0.154^c^	0.224^c^	0.524^b^	0.496^b^	0.0272	0.0008
Spleen	0.023	0.025	0.022	0.038	0.043	0.0023	0.0881
Thymus	0.323	0.306	0.277	0.269	0.383	0.0291	0.0963

MOLM, *Moringa oleifera* leaf meal; SEM, standard error of mean.

a–c Means in the same row followed by different letters are significantly different at (p<0.05).
